# Regular Health Checks: Cross-Sectional Survey

**DOI:** 10.1371/journal.pone.0033694

**Published:** 2012-03-30

**Authors:** Christian Grønhøj Larsen, Karsten Juhl Jørgensen, Peter C. Gøtzsche

**Affiliations:** Nordic Cochrane Centre, Rigshospitalet and University of Copenhagen, Copenhagen, Denmark; Agenzia di Sanita Pubblica Regione Lazio, Italy

## Abstract

**Objective:**

To investigate whether Danish providers of general health checks present a balanced account of possible benefits and harms on their websites and whether the health checks are evidence-based.

**Methods and Design:**

Cross-sectional study. The search engines Google and Jubii (Danish) were in July and August 2009 used to identify 56 websites using Danish search terms for “health check” and “health examination”. The content of the websites were evaluated using a checklist with 15 officially recommended information items. All tests offered through the websites were registered. The evidence for tests offered through at least 10% of the websites was identified in structured searches using PubMed and The Cochrane Library.

**Results:**

We found 36 different tests on 56 websites offering health checks. Twenty one tests were offered on at least 10% of the websites. Seventeen (81%) of these tests were unsupported by evidence, or there was evidence against them for screening purposes. We found evidence supporting screening using body-mass-index, blood pressure, cholesterol, and faecal occult blood testing. None of the websites mentioned possible risks or harms. The websites presented a median of 1 of the 15 information items; the highest number from any provider was 2.

**Conclusions:**

Information from Danish providers of health checks was sparse and tests were often offered against existing evidence or despite lack of evidence. None of the included websites mentioned potential risks or harms.

## Introduction

Regular health checks of healthy individuals are intended to identify risk factors and early signs of disease, preventing future illness through early intervention. This strategy may seem immediately appealing but in some cases, potential harms can outweigh the potential benefits.

Quite often, healthy people with common risk factors would not have developed the disease intended to prevent, even without screening. In these cases, the identification of risk factors represents overdiagnosis, which may lead to unnecessary additional diagnostic workups with possible complications. It may also increase the use of medication, which will usually only be harmful in overdiagnosed people. Identification of risk factors may also cause psychological stress, with a negative impact on quality of life.

Health checks are an unregulated market, which adds to the complexity. Private practices are not obliged to provide further diagnostic workups, treatment, or follow-up tests when they uncover risk factors. The expenses associated with this can drain resources from public health care that could perhaps be used for a better purpose. It is a common misconception that screening programmes and early treatment will generally save money in the long run, and some screening programmes are very costly [1].

Some trials of health checks have found beneficial effects on risk factors for cardiovascular disease [2] [3] [4], but trials with morbidity and mortality as outcomes have not been convincing. An American randomized trial published in 1986 evaluated annual health checks through 16 years and included 10,713 men and women aged 35–54 years. It found an effect on mortality related to pre-specified potentially postponable causes, but did not find any difference in overall mortality or hospitalization rate. A British trial from 1977 evaluated two general health checks of 7,229 men and women aged 40–64 years and reached similar results after 9 years of follow-up. A Swedish trial from 1998 randomized 3,064 men and women to a single general health check and 29,122 to a control group and did not find an effect on mortality after 22 years of follow-up. The health checks provided in these trials were all rather extensive (table 1).

**Table 1:** Content of health checks in randomised trials.

Friedman et al. [2]	The South-East London Screening Study Group. [3]	Theobald et al. [4]
A medical questionnaire, blood pressure, electrocardiogram, audiography, visual test, tonometry, spirometry, chest X-ray, mammography for women age 48 years and older, urine analysis, blood tests incl. haematology, serum chemistry panels, gynecological examination, Papanicolaou cervical smear, sigmoidoscopy for persons over 40 years, and a follow-up visit to a physician for test results.	A self-administered symptoms questionnaire, interviewer-administered questions, body proportions, weight and height, visual test, audiometry, chest X-ray, lung function tests, electrocardiogram, blood pressure, blood tests (Haemoglobin, packed cell volume, blood urea, random blood sugar, protein-bound iodine, cholesterol, uric acid), stool for occult blood and basic physician examination.	A postal questionnaire, blood tests, electrocardiogram, exercise tests, psychological tests and eye and dental examinations.

The health check industry is growing fast in many countries. In Denmark, this is partly because new legislation provided tax-exemption for private health insurance. We studied the websites from Danish providers of general health checks and quantified the tests and the information offered. We also did literature searches to see if the included tests were supported by evidence.

## Materials and Methods

We included websites that advertised screening for several diseases and risk factors as a package. Websites offering screening for single specific diseases or non-scientific tests (e.g. iris analysis) were excluded.

In Denmark, 60% of the population use the Internet to gather information on health issues [5]. We therefore used simple searches to locate providers of health checks that potential customers would find easily. Danish websites were located using Google and the Danish search engine Jubii, using Danish terms for health checks (Sundhedstjek, Sundhedscheck, Sundhedsundersøgelse, Helbredstjek, Helbredscheck and Helbredsundersøgelse). CGL browsed the first 10 pages of results retrieved for each search term, 120 pages in total. These were saved as PDF documents. When a search term is entered into the Google search bar, targeted advertising of websites appears in the top and the right hand side of the page. These websites were also included.

We used a pre-specified checklist of 15 information items to study whether the information presented on websites by providers of health checks gave a balanced account of the possible benefits and harms of the tests offered (table 2). These 15 items are recommended information items about screening healthy people from the World Health Organization and The Danish National Board of Health [6]. Overall, they address which diseases are being screened for, the possibility of a false positive or false negative result, the accuracy of the test to diagnose a person as being ill or healthy, and information on the number of people being overdiagnosed and overtreated. In addition, the websites were searched for information on how the test answers would be communicated and how a possible illness was to be treated.

**Table 2:** Information items on websites.

	Websites (n = 56)	
Information	Absolute number	Pct (%)
Presentation of test answers (personal meeting, written report, or both)	39	70
Diseases screened for	6	11
Other relevant information on test or results	3	5

No websites quantified the effect of screening, or mentioned the risk of false negative results, false positive results, overdiagnosis, overtreatment, lifetime risk for developing the disease tested for, sensitivity, specificity, negative predictive value, positive predictive value, or the psychological stress related to false positive results and or treatment of early disease.

Finally, CGL reviewed the literature for evidence about the individual tests, restricted to those that were represented on more than 10% of the websites. The Cochrane Library and PubMed were searched for a specific illness, condition or test, e.g. “hypertension” (condition) or “blood pressure” (test), combining this with either “asymptomatic” or “screening.” All reviews and guidelines identified by the searches, and relevant studies indentified in the reference lists, were retrieved.

## Results

We identified 56 websites from providers of health checks; 53 were commercial organizations (20 run by doctors and 33 run by other medical personnel) and 3 from non-profit organizations (The Danish Heart Organization, Hørsholm Municipality and Hjørring Municipality).

The websites offered 36 different screening tests, and 21 of these were represented on more than 10% of the websites (Table 3). Of these 21 tests, we found recommendations *against* using the test for screening purposes for 48% (n = 10) of the tests and lacking or inconclusive evidence for another 33% (n = 7) of the tests, in total 81% (n = 17).

**Table 3:** Tests provided as part of general health checks in Denmark.

	Websites n = 56	
Screening name or type	Absolute	Pct
Cancer tests		
Blood in faeces	8	14
PSA	7	13
**Meeting with health professionel**		
Clinical examination by doctor	13	23
Conversation or questionnaires	26	46
Vision and hearing	7	13
**Heart and lung measures**		
Blood pressure	46	82
Electrocardiogram/ECG (rest)	13	23
Electrocardiogram/ECGE (exercise)	16	29
Lung function test/spirometry	18	32
**Radiology**		
X-ray thorax	5	9
**Urinary dipstick**	13	23
**Lab tests**		
Blood sugar	47	84
Kidney tests	12	21
Lipids (Cholesterol)	49	88
Liver tests (ALAT or ASAT)	10	18
Infection parameters (CRP, Sedimentation rate or white blood cells)	6	11
Electrolytes	5	9
Clinical biochemistry tests referred to as “Blood/Lab” tests (not specified)	10	18
Haemoglobin/Blood pct	12	21
Thyroidea (TSH)	6	11
Lung tests (PO2 or CO in blood)	5	9
**Body measures**		
Body composition (e.g. fat percentage)	35	63
Weight, height, waist (e.g. Body mass index)	43	77
Fitness test/rating	24	43
**Unknown tests**		
Strength and suppleness	4	7
Unknown fitness tests (e.g. physical age or oxidative stress)	8	14

The following tests were represented on less than 3 web sites: Rectoscopy, Vaginal smear, Heart blood tests (Pro-BNP), Echocardiogram, Pancreas (alfa amylase), Mammography, Ultrasound of abdomen, Unspecified/Unknown (eg. Bone marrow test), Virus parameters (HIV, Hepatitis B/C) and Vitamins (25-OH-D-Vit.)

The sites included a median of 1 information item out of the 15 recommended items; the highest number included was 2. The most common information item (70% of the sites, n = 56) was how the test result was provided (a written report, personal meeting, or both).

None of the websites quantified the expected benefit of screening or mentioned the harms. Further, there was no information on the risk of false negative results, the risk of false positive results, the level of overdiagnosis, the level of overtreatment, the lifetime risk for developing the disease tested for, the sensitivity, the specificity, the negative predictive value, the positive predictive value, or the psychological stress related to false positive results (table 2).

Evidence existed to support screening asymptomatic people for body-mass-index [7], [8], blood pressure [9], cholesterol [7], and faecal occult blood tests [10].

There was inconclusive evidence on screening for thyroid-stimulating hormone (TSH) and it was unclear whether treatment will improve the quality of life in otherwise healthy asymptomatic adults with abnormal TSH levels [11]. No studies have demonstrated hearing screening to improve hearing function. We found a recommendation for screening older people but a specific age cut-off is unclear [7] and a randomised controlled trial found no affect of screening for hearing loss [12]. Vision screening in adults older than 65 years of age is recommended [7] but direct evidence shows no benefit and the U.S. Preventive Tasks Force conclude that more research is needed [13]. For these reasons we found the evidence inconclusive for vision and hearing screening in adults.

No studies or recommendations were found addressing the benefits and harms of screening asymptomatic people for alanine aminotransferase (ALAT), aspartate aminotransferase (ASAT), infection parameters (C-reactive protein, sedimentation rate or white blood cells), fitness ratings or fat percentages.

Evidence, recommendations, or both, were found *against* offering a general physical examination [7], screening for prostate cancer with prostate specific antigen-testing [14] [15], anaemia blood tests [7], coronary heart disease with electrocardiograms or exercise electrocardiograms [7], [16], diabetes with blood glucose [7], [17], [18], kidney disease with serum creatinine [19], urinary dipstick [7], [20], [21], ‘lab tests’ [7], and spirometry [22] for asymptomatic persons. The results of our review of the evidence are summed up in Table 4.

**Table 4:** Overview of screening tests.

Screening test	Health checks offering test n = 56	Recommendation
Cholesterol	88%	Men>age 34Women >age 44 [7]
Blood glucose	84%	Recommendation against screening [7] [17] [18]
Blood pressure	82%	Screening recommended [9]
Body mass index	77%	Screening recommended [7] [8]
Body composition (fat percentage)	63%	No studies or recommendations found
Fitness rating	43%	No studies or recommendations found
Lung function test	32%	Recommendation against screening [22]
ECG	29%	Recommendation against screening [7] [16]
ECGE	23%	Recommendation against screening [7] [16]
Physical examination	23%	Recommendation against screening [7]
Urinary dipstick	23%	Recommendation against screening [7] [20] [21]
Haemoglobin	21%	Recommendation against screening [7]
Kidney test	21%	Recommendation against screening [19]
ALAT/ASAT	18%	No studies or recommendations found
Unspecified “blood/lab” testing	18%	Recommendation against screening [7]
Faecal occult test	14%	Screening recommended [10]
PSA	13%	Recommendation against screening [14] [15]
Hearing	13%	Inconclusive evidence [12]Recommendation for “older adults” [7]
Vision	13%	Inconclusive evidence [13]Recommendation for adults>age 65 [7]
Infection parameters	11%	No studies or recommendations found
TSH	11%	Inconclusive evidence [11]

## Discussion

The information on Danish websites from providers of health checks was sparse and severely biased in favour of health checks. None of the websites quantified the expected benefit of screening or provided information on possible risks and harms. The majority of the tests offered (81%) were either recommended against, or there was a lack of evidence or recommendations.

Health checks may be of value to individuals seeking reassurance on their state of health but suppliers must provide information that no screening test is perfect and may result in important harms. A negative result is no guarantee that a person is healthy, or that they will stay healthy. Potential participants must be also be informed that most tests were often developed for diagnostic purposes, not for screening, and that this would be expected to decrease their reliability substantially. Potential participants must also be informed that the results of the tests are often evaluated by a nurse or other staff who are not trained to diagnose an illness or prescribe treatment. This information was not presented on any website that advertised health checks by nurses or other healthcare personnel.

For all screening tests, the low prevalence of disease in a healthy population and a different spectrum of disease severity among those who actually have the disease, will cause a reduction in the predictive value of a positive test result. This reduction increases the risk that healthy individuals will receive a false-positive result leading to unnecessary follow-up tests and overtreatment. The economic consequences of screening tests with a large proportion of false-positives may therefore be considerable. These costs are covered by the public healthcare system, which may lead to a suboptimal allocation of resources.

An additional problem is that pre-symptomatic treatment might not improve the long-term mortality or morbidity compared to symptomatic treatment, but can have additional side effects and will also lead to more years being a patient rather than staying healthy.

Information on websites from providers of general health checks was sparse, the expected benefit of screening was not quantified, and the risks and harms of the screening tests were not described on any website. Eighty-one percent of the tests included in the health checks were either recommended against for screening purposes, or there were lacking evidence or recommendations for their use as screening tools.

We believe it is unethical to advertise health checks that lack evidence to support them and indefensible to promote screening tests when there is evidence against using the tests for screening purposes. It is also counter to Danish health legislation to provide information about health interventions without presenting a balanced account of the benefits and harms, even if the harms are rare.

We call for better regulation of this growing industry.

## References

[1] Raffle A, Gray M (2007). Screening: evidence and practice.

[2] Friedman GD, Collen MF, Fireman BH (1986). Multiphasic Health Checkup Evaluation: a 16-year follow-up.. J Chronic Dis.

[3] Holland WW, Creese AL, D'Souza MF, Partridge JRJ, Woodall HJT (1977). A controlled trial of multiphasic screening in middle-age: results of the South-East London Screening Study. The South-East London Screening Study Group.. Int J Epidemiol.

[4] Theobald H, Bygren LO, Carstensen J, Hauffman M, Engfeldt P (1998). Effects of an assessment of needs for medical and social services on long-term mortality: a randomized controlled study.. Int J Epidemiol.

[5] Voss H, Ravn BL (2007). Danskernes brug af sundhedsydelser på internettet.. Ugeskr Laeger.

[6] Kjøller M, Juel K, Kamper-Jørgensen F (2007). Folkesundhedsrapporten.

[7] Health care guidelines (2009). Preventive services for adults.. Institute for Clinical Systems Improvement.

[8] McTigue KM, Harris R, Hemphill B, Lux L, Sutton S (2003). Screening and interventions for obesity in adults: summary of the evidence for the U.S. Preventive Services Task Force.. Ann Intern Med.

[9] Lewington S, Clarke R, Qizilbash N, Peto R, Collins R (2002). Age-specific relevance of usual blood pressure to vascular mortality: a meta-analysis of individual data for one million adults in 61 prospective studies.. Lancet.

[10] Hewitson P, Glasziou P, Irwig L, Towler B, Watson E (2007). Screening for colorectal cancer using the faecal occult blood test, Hemoccult.. Cochrane Database Syst Rev.

[11] Helfand M (2004). Screening for subclinical thyroid dysfunction in nonpregnant adults: a summary of the evidence for the U.S. Preventive Services Task Force.. Ann Intern Med.

[12] Karlsmose B, Lauritzen T, Engberg M, Parving A (2001). A randomised controlled trial of screening for adult hearing loss during preventive health checks.. Br J Gen Pract.

[13] Chou R, Dana T, Bougatsos C (2009). Screening older adults for impaired visual acuity: a review of the evidence for the U.S. Preventive Services Task Force.. Ann Intern Med.

[14] Andriole GL, Crawford ED, Grubb RL, Buys SS, Chia D (2009). Mortality results from a randomized prostate-cancer screening trial.. N Engl J Med.

[15] Schroder FH, Hugosson J, Roobol MJ, Tammela TL, Ciatto S (2009). Screening and prostate-cancer mortality in a randomized European study.. N Engl J Med.

[16] Fowler-Brown A, Pignone M, Pletcher M, Tice JA, Sutton SF (2004). Exercise tolerance testing to screen for coronary heart disease: a systematic review for the technical support for the U.S. Preventive Services Task Force.. Ann Intern Med.

[17] Norris SL, Kansagara D, Bougatsos C, Fu R (2008). Screening adults for type 2 diabetes: a review of the evidence for the U.S. Preventive Services Task Force.. Ann Intern Med.

[18] Woolthuis EPK, Grauw WJD, Laar FAVd, Akkermans RP (2009). Screening for type 2 diabetes mellitus (Protocol).. Cochrane Database of Systematic Reviews.

[19] Wu I, Parikh CR (2008). Screening for kidney diseases: older measures versus novel biomarkers.. Clin J Am Soc Nephrol.

[20] Lin K, Fajardo K (2008). Screening for asymptomatic bacteriuria in adults: evidence for the U.S. Preventive Services Task Force reaffirmation recommendation statement.. Ann Intern Med.

[21] Ruttimann S, Clemencon D (1994). Usefulness of routine urine analysis in medical outpatients.. J Med Screen.

[22] Lin K, Watkins B, Johnson T, Rodriguez JA, Barton MB (2008). Screening for chronic obstructive pulmonary disease using spirometry: summary of the evidence for the U.S. Preventive Services Task Force.. Ann Intern Med.

