# Correction: Regular Health Checks: Cross-Sectional Survey

**DOI:** 10.1371/annotation/db021c13-fba1-4ad3-8711-9bf7c00fda6b

**Published:** 2012-09-12

**Authors:** Christian Grønhøj Larsen, Karsten Juhl Jørgensen, Peter C. Gøtzsche

Three references are missing from the published article. These references are:

Imperial Cancer Research Fund OXCHECK Study Group (1995) Effectiveness of health checks conducted by nurses in primary care: final results of the OXCHECK study. BMJ 310: 1099-1104.

Family Heart Study Group (1994) Randomised controlled trial evaluating cardiovascular screening and intervention in general practice: principal results of British family heart study. BMJ 308: 313-320.

Engberg M, Christensen B, Karlsmose B, Lous J, Lauritzen T (2002) General health screenings to improve cardiovascular risk profiles: a randomized controlled trial in general practice with 5-year follow-up. J Fam Pract 51: 546-552.

These three references should replace the citations [2], [3], and [4] cited in the text in the first sentence of the fourth paragraph of the Introduction, "Some trials of health checks have found beneficial effects on risk factors for cardiovascular disease..."

Citations [2], [3], and [4], belong elsewhere in the same paragraph:

An American randomized trial published in 1986 evaluated annual health checks through 16 years and included 10,713 men and women aged 35–54 years [2]. It found an effect on mortality related to pre-specified potentially postponable causes, but did not find any difference in overall mortality or hospitalization rate. A British trial from 1977 evaluated two general health checks of 7,229 men and women aged 40–64 years and reached similar results after 9 years of follow-up [3]. A Swedish trial from 1998 randomized 3,064 men and women to a single general health check and 29,122 to a control group and did not find an effect on mortality after 22 years of follow-up [4]. The health checks provided in these trials were all rather extensive (table 1). 

